# Yiyi Fuzi Baijiang Decoction Alleviates Ulcerative Colitis Partly by Regulating TLR4-Mediated PI3K/Akt and NF-*κ*B Pathways

**DOI:** 10.1155/2022/8780514

**Published:** 2022-02-15

**Authors:** Liping Chen, Chunyu Zhang, Jingjing Cao, Ge Bei, Xiaoyu Wang, Zhiwei Miao

**Affiliations:** ^1^Department of Gastroenterology, Zhangjiagang TCM Hospital Affiliated to Nanjing University of Chinese Medicine, Suzhou 215600, China; ^2^Department of TCM Classics, Wuxi TCM Hospital Affiliated to Nanjing University of Chinese Medicine, Wuxi 214072, China

## Abstract

Yiyi Fuzi Baijiang Decoction (YFBD), an ancient prescription developed by the ancient Chinese physician, Zhang Zhongjing, has shown remarkable effects in treating ulcerative colitis (UC). However, there are few studies on its mechanism. This study was designed to explore the potential mechanism of YFBD in treating UC. The principal ingredients of YFBD were analyzed using high-performance liquid chromatography (HPLC). Dextran sulfate sodium- (DSS-) induced mice and lipopolysaccharide- (LPS-) stimulated RAW264.7 cells were used in the study. The body weight and disease activity index (DAI) of mice were recorded and analyzed for 10 days. After sacrifice, the colonic tissues were harvested. The colon length was measured, and the histopathological changes were observed by hematoxylin and eosin staining. The levels of inflammatory cytokines in mice colons and RAW246.7 cells were determined by real-time quantitative PCR and immunofluorescence. The effects of YFBD on the TLR4-mediated PI3K/Akt and NF-*κ*B pathways were determined by western blot analysis. HPLC identified five compounds in YFBD: chlorogenic acid, caffeic acid, benzoylmesaconine, benzoyl aconitine, and quercetin. YFBD alleviated weight loss, colon shortening, and colonic histopathological lesion in mice. Meanwhile, it decreased the DAI and histological score of mice with UC. In addition, YFBD remarkably decreased the levels of interleukin- (IL-) 6, IL-1*β*, and tumor necrosis factor (TNF)-*α* in the colons of DSS-induced mice and LPS-stimulated RAW246.7 cells. Furthermore, the expression of key proteins in TLR4-mediated PI3K/Akt and NF-*κ*B pathways significantly decreased with YFBD treatment. In conclusion, YFBD had protective effects on mice with UC, which was in part related to its anti-inflammatory effects and downregulation of TLR4-mediated PI3K/Akt and NF-*κ*B pathways.

## 1. Introduction

Ulcerative colitis (UC) is characterized by chronic nonspecific inflammation with recurring cycles of tissue destruction and repair. The highest incidence of UC was reported in Europe and North America (24.3 and 19.2 per 100,000, resp.) [1]. The morbidity follows an ascending trend, especially in newly industrialized countries [2–4]. Currently, drugs for UC, including aminosalicylates, corticosteroids, immunomodulators, and tumor necrosis factor (TNF)-*α* inhibitor, have achieved favorable efficacy in clinical practice. Sulfasalazine is one kind of traditional aminosalicylate products, which serves as first-line therapy on UC and is beneficial to most patients with mild-to-moderate, left-sided colitis [5]. However, these agents have limitations, including severe side effects, even tumorigenesis, and high costs [6]. Therefore, more effective, less toxic, and cheaper drugs need to be developed to benefit patients with UC.

Traditional Chinese medicine (TCM) was demonstrated to have the therapeutic potential to treat UC [7, 8]. Yiyi Fuzi Baijiang Decoction (YFBD), a formula originally presented in the Chinese medical classic *Shang Han Lun*, can be used to treat UC based on TCM theory. YFBD showed positive effects among patients [9, 10]. It has three components: *C. lacryma-jobi L.* (Yi Ren), *Radix Aconiti Lateralis Preparata* (Fuzi), and *Patrinia scabiosaefolia* Fisch. (Bai Jiang Cao). *C. lacryma-jobi* L. could relieve the symptoms of UC by regulating the proinflammatory mediators and improving the microbial community structure in the gut [11, 12]. *Radix Aconiti Lateralis Preparata* had far-ranging pharmacological activities; it displayed anti-inflammatory, antitumor, and immunoregulatory effects and influenced energy metabolism [13]. It also reduced inflammation in mice with UC partly by inhibiting the NF-kB pathway [14]. The extracts in *P. scabiosaefolia* Fisch. could reduce inflammatory responses in extensive models, such as UC, acute pancreatitis, and focal cerebral ischemia-reperfusion models [15]. Previous studies provided clues and evidence on the anti-inflammatory effects of YFBD. However, the underlying mechanism remains to be explored.

TLR4-mediated PI3K/Akt and NF-*κ*B pathways closely relate to inflammatory responses of UC. TLR4, as a member of the Toll-like receptor family, can mediate inflammatory responses [16]. Previous studies confirmed that TLR4 expression was upregulated in patients with UC [17, 18]. TLR4 recognized lipopolysaccharide (LPS) and initiated intracellular signaling, such as PI3K/Akt and NF-*κ*B pathways [19–22]. The PI3K/Akt pathway was generally considered to be linked with the cellular processes of metabolism, proliferation, growth, and survival. However, current evidence suggests that the pathway participates in the pathogenesis of UC based on its extensive regulation of inflammation, apoptosis, and immune responses [23–25]. NF-*κ*B is involved in gene expression of inflammatory cytokines [26]. Studies showed that persistent activation of NF-*κ*B signaling could be detected in patients with UC and murine UC models [7, 18]. Therefore, it was inferred that TLR4-mediated PI3K/Akt and NF-*κ*B pathways were critical to the development of UC and were probably promising therapeutic targets.

Consequently, considering the pharmacological study on the ingredients of YFBD and the mechanism of UC, it was hypothesized that YFBD could alleviate UC by controlling inflammation, which was partly realized by regulating TLR4-mediated PI3K/Akt and NF-*κ*B pathways. In the research, the levels of inflammatory cytokines and key proteins in these pathways were measured, and the possible mechanism behind YFBD treating UC was clarified for the first time.

## 2. Materials and Methods

### 2.1. Drugs and Antibodies

Dextran sulfate sodium (DSS, 36–50 kDa) was purchased from MP Biomedicals (CA, USA). Primary antibodies against p-PI3K (#4228), Akt (#4691), and p-Akt (#4060) were purchased from Cell Signaling Technology (MA, USA). Primary antibodies against TLR4 (ab13867), NF-*κ*B/p65 (ab32536), p-NF-*κ*B/p65 (ab28856), I*κ*B*α* (ab32518), *β*-actin (ab6276), IL-6 (ab233706), IL-1*β* (ab216995), TNF-*α* (ab215188), as well as HRP-conjugated goat anti-rabbit/mouse IgG (ab150077, ab205719) and goat anti-rabbit IgG H&L Alexa Fluor 488/555(ab150077, ab150078), were purchased from Abcam (MA, USA). Primary antibody against p-I*κ*B*α* (AP0707) was purchased from ABclonal Biotechnology (Wuhan, China). Lipopolysaccharide (LPS) was purchased from Sigma (MO, USA). The three medicinal herbs in YFBD (*C. lacryma-jobi* L., *Radix Aconiti Lateralis Preparata*, and *P. scabiosaefolia* Fisch.) were purchased from Beijing Tong Ren Tang Co., Ltd. (Suzhou, China). Sulfasalazine (SASP) was purchased from Shanghai Sine Tianping Pharmaceutical Co., Ltd. (Shanghai, China).

### 2.2. Preparation of Drugs

Raw herbs (shown in Table 1) were immersed in the 10× volume of distilled water for 30 min, boiled at 100°C for another 30 min, cooled to room temperature, and filtered through a 200-mesh filter to make YFBD raw decoction. Drug sediments were reserved for the second decoction using an 8× volume of distilled water, and the solution was filtered again. Both batches of the filtrate were mixed, concentrated to 2 g/mL and 1 g/mL decoction, respectively (calculated with raw herbs), and stored at 4°C for the intragastric administration of mice and high-performance liquid chromatography (HPLC) analysis. To prepare YFBD powder acting on cells, 50 mL YFBD (1 g/mL) was freeze-dried by a LAB-1A-50E freeze dryer (Biocool, Beijing, China) into 1.84 g brown powder.

### 2.3. Standardization of YFBD

The standards including chlorogenic acid (C8960; purity ≥ 98%), caffeic acid (C8990; purity ≥ 99%), and quercetin (SQ8030; purity ≥ 98%) were purchased from Solarbio (Beijing, China). The standards including benzoylmesaconine (T6S1885; purity: 99.59%) and benzoyl aconitine (T6S1880; purity: 99.78%) were purchased from Topscience (Shanghai, China). The concentrations of the main compounds in YFBD were determined using HPLC [27, 28] with minor modifications. Briefly, a Shim-pack VP-ODS C_18_ column ((250 × 4.6 mm^2^, 5.0 *μ*m) was used for chromatography separation. The mobile phase consisted of 0.2% formic acid and 10 mM ammonium acetate (A) and acetonitrile (B). The gradient elution program was as follows: 0⟶12 min, 5% B; 12⟶28 min, 5% B⟶30% B; 28⟶33 min, 30% B; 33⟶35 min, 30% B⟶5% B; 35⟶40 min, 5% B. The wavelength was set at 230 nm because this wavelength could detect all the compounds with acceptable sensitivity.

### 2.4. Animals

Fifty male C57BL/6 mice (age: 6 weeks; weight: 18–20 g) were purchased from Suzhou JOINN Clinical Co., Ltd. with license No. SYXK (Su) 2017–0043. They were housed in a controlled environment at a temperature (24 ± 1°C) under a standard light-dark cycle with free access to food and drink. The animal research was conducted conforming to the protocol approved by the Institute of Animal Care Committee of Zhangjiagang TCM Hospital.

### 2.5. Grouping and Induction of UC

**Figure 1:**
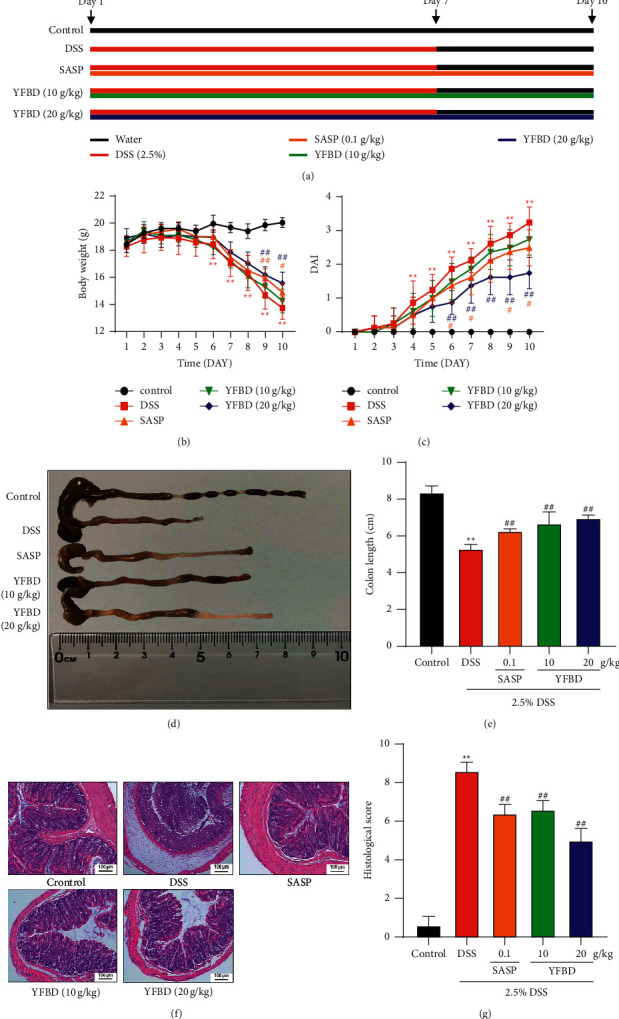
YFBD attenuated DSS-induced acute colitis. (a) Animal experiment design (10/group). (b) Changes in body weight. (c) Evaluation of DAI. (d) Representative images of colons. (e) Effect of YFBD on colon length. (f) Photographs of H&E-stained colon sections (magnification: ×100). (g) Assessment of the histological scores. Data are presented as mean ± SEM (*n* = 8). ^*∗∗*^*P* < 0.01 vs. control; ^#^*P* < 0.05; ^##^*P* < 0.01 vs. DSS.

The mice were randomly divided into five groups (*n* = 10): control group (sterile water), colitis group (2.5% DSS), SASP group (2.5% DSS + SASP 0.1 g/kg), YFBD low-dose group (2.5% DSS + YFBD 10 g/kg), and YFBD high-dose group (2.5% DSS + YFBD 20 g/kg). The experiment was started after seven days of adaptive feeding. The mice in the control group received sterile water for 10 days. The mice in the DSS, SASP, and YFBD groups were given 2.5% DSS for seven days to introduce colitis models, following which they were fed sterile water for three days. Meanwhile, the mice in treatment groups were orally administered with SASP (0.1 g/kg), YFBD (10 g/kg), and YFBD (20 g/kg) for 10 days. The mice in control and DSS groups were given the equal volume of distilled water by gavage. Animal equivalent doses were converted from human doses based on the body surface area [29]. After sacrifice, mice colons were cut into three sections for further research. The animal experimental design is shown in Figure 1(a).

### 2.6. Disease Activity Index and Histological Evaluation

During drug administration and modeling, the body weight, stool characteristics, and bleeding were observed and recorded daily according to scoring system referred earlier [30]. The distal colonic tissues were fixed with 10% formalin, embedded in paraffin, sliced into four-µm thick sections, and mounted on microscope slides. The slices were tinted with hematoxylin and eosin (H&E) and then photographed under a microscope. The colonic pathology was scored based on the modified histology scoring system as described previously [31].

### 2.7. Cell Culture and Treatment

RAW264.7 macrophages were obtained from the Cell Bank of the Chinese Academy of Sciences (Shanghai, China). The cells were cultured in high-glucose DMEM (Hyclone, UT, USA) supplemented with 10% FBS (Biological Industries, Israel), 100 U/mL penicillin, and 0.1 mg/mL streptomycin (NCM Biotech, Suzhou, China) in the presence of humidified 5% CO_2_ at 37°C. The cells were seeded at 10^5^/mL for 24 h, followed by incubation with different concentrations of YFBD for 2 h, and then stimulated with LPS (1 *μ*g/mL) for another 24 h.

### 2.8. Small Interfering RNA (siRNA) Transfection

The TLR4 siRNA target sequence and negative control siRNA sequence were purchased from Santa Cruz Biotechnology (CA, USA). Briefly, RAW 264.7 cells were cultured in 6-well plates (5 × 10^5^ cells/well). When the cell density reached 40–50%, NC siRNA or TLR4 siRNA were transfected into cells with Lipofectamine 3000 (Invitrogen, USA) according to the manufacturer's protocol. After a 24-hour transfection period, the cells were treated for 2 h with or without YFBD and then exposed to LPS (1 *μ*g/ml) for 24 hours, followed by other analyses.

### 2.9. Cell Viability Assay

RAW 264.7 macrophages (10^4^/well) were inoculated in 96-well plates for 24 h and then cultured with multiple concentrations of YFBD (0, 6.25, 12.5, 25, 50, 100, 200, and 400 *μ*g/mL) for 24 h. Subsequently, 10 *μ*L of cell counting kit-8 (CCK-8; Dojindo Co., Kumamoto, Japan) was added to each well and incubated for another 1 h. The optical density (OD) values were measured at 450 nm (BioTek, VT, USA).

### 2.10. RNA Isolation and RT-qPCR Assay

Total RNA was isolated from colon tissues and RAW 264.7 cells with TRIzol regent (Invitrogen, CA, USA). The RNA concentration was examined using a NanoDrop spectrophotometer (Thermo, MA, USA), and then RNA was reverse-transcribed to cDNA using an Applied Biosystems thermal cycler (Thermo, MA, USA). Afterwards, qRT-PCR was performed on a LightCycler 96 real-time PCR detection system (Roche, BW, Germany) following the instructions of SYBR Green (Thermo). Relative mRNA expression was calculated using the comparative Ct method (2^−△△Ct^). The primers were purchased from Sangon Biotech (Shanghai, China), and the sequences are listed in Table 2.

### 2.11. Immunofluorescence Staining

Immunofluorescence staining was performed on colon paraffin sections with 4 *μ*m thickness. Briefly, the sections were boiled in citric acid buffer (Beyotime, Shanghai, China) for 20 min. After washing with PBS (Beyotime) three times, the sections were immersed in Triton X-100 (Beyotime) and subsequently blocked in 5% serum. Afterwards, they were incubated with primary antibodies at 4°C overnight. The colon sections were washed and coincubated with corresponding fluorescence-conjugated secondary antibodies in the dark, followed by staining with DAPI (Beyotime). Finally, the images of colon sections were captured under an epifluorescence microscope (Olympus, U-RFL-T, Japan).

### 2.12. Western Blot Analysis

A Protein Quantification Kit (BCA Assay; Thermo) was used to quantify the protein concentrations of extracts from colon tissues and cultured cells. The protein samples were separated by SDS-PAGE (Beyotime) and transferred onto PVDF membranes (Millipore Corp.; MA, USA), which were then blocked with 5% bovine serum albumin (Thermo) in TBST (Beyotime). The membranes were incubated with the primary antibodies against TLR4 (1 : 500), p-PI3K (1 : 1000), Akt (1 : 1000), p-Akt (1 : 2000), NF-*κ*B/p65 (1 : 5000), p-NF-*κ*B/p65 (1 : 1000), I*κ*B*α* (1 : 5000), p-I*κ*B*α* (1 : 1000), and *β*-actin (1 : 5000) and then rinsed with TBST three times, followed by coincubation with the corresponding secondary antibodies (1 : 5000). Finally, the chemiluminescence signals were detected, and the band intensity was quantified using ImageJ software.

### 2.13. Statistical Analysis

Each experiment was performed at least three times, and all data were expressed as mean ± SEM. The significance of differences was determined using one-way analysis of variance with Tukey's multiple comparison test using SPSS software 21.0. *P* values less than 0.05 indicated statistically significant differences.

## 3. Results

### 3.1. HPLC Analysis of the Main Constitutes in YFBD Extracts

**Figure 2:**
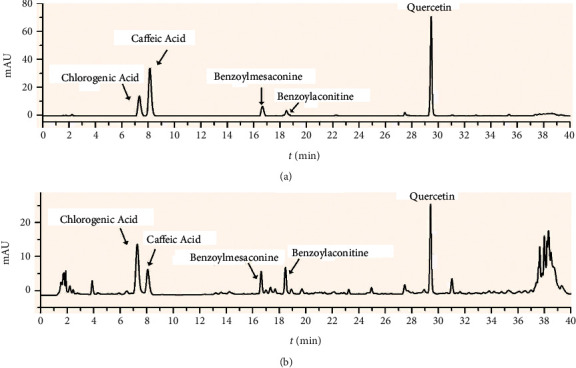
HPLC analysis of the main chemicals of YFBD recorded at 230 nm. (a) Solution of standards. (b) Solution of YFBD.

The chromatographs of the standard mixtures and the YFBD are shown in Figure 2. The contents of these five chemicals in YFBD (1 g/mL) were 106.2, 25.3, 62.5, 31.7, and 18.4 *μ*g/mL for chlorogenic acid, caffeic acid, benzoylmesaconine, benzoyl aconitine, and quercetin, respectively.

### 3.2. YFBD Alleviated DSS-Induced Colitis

Distinct body weight loss was observed from day 6 (*P* < 0.01), and the DAI score significantly increased on day 4 in the DSS group compared with the control group (*P* < 0.01). The body weight increased, and the DAI score dropped significantly in YFBD (20 g/kg) and SASP groups compared with the DSS group (*P* < 0.05 or 0.01; Figures 1(b) and 1(c)). Besides, DSS shortened the colon length significantly (*P* < 0.01; Figures 1(d) and 1(e)). However, the colon lengthened in the YFBD (10, 20 g/kg) and SASP groups compared with the DSS group (*P* < 0.01; Figures 1(d) and 1(e)). Furthermore, H&E staining showed that DSS administration induced severe colonic damage, such as mucosal ulceration and crypt damage along with leukocyte cell infiltration. However, treatment with YFBD (10, 20 g/kg) and SASP markedly attenuated colonic damage (Figure 1(f)). Moreover, the histological score increased appreciably in the DSS group (*P* < 0.01), while it declined in the YFBD (10, 20 g/kg) and SASP groups compared with the DSS group (*P* < 0.01; Figure 1(g)).

### 3.3. YFBD Decreased the Levels of Inflammatory Cytokines in Mice with UC

**Figure 3:**
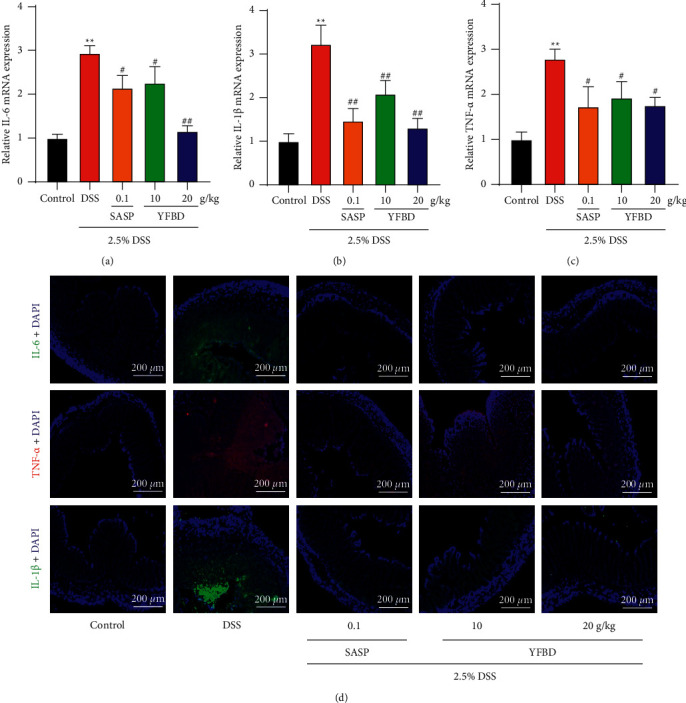
YFBD decreased the inflammatory cytokines in colons of mice with DSS-induced colitis. (a) mRNA level of IL-6. (b) mRNA level of IL-1*β*. (c) mRNA level of TNF-*α*. (d) Colon sections were stained with DAPI (blue) and IL-6 (green), IL-1*β* (green), and TNF-*α* (red) and observed under a fluorescence microscope. Data are presented as mean ± SEM (*n* = 3). ^*∗∗*^*P* < 0.01 vs. control; ^#^*P* < 0.05; ^##^*P* < 0.01 vs. DSS.

A marked increase in mRNA levels of IL-6, IL-1*β* and TNF-*α* was observed after DSS induction in the control group (*P* < 0.01; Figures 3(a)–3(c)). However, the mRNA levels of the cytokines decreased in the YFBD (10 and 20 g/kg) and SASP groups compared with the DSS group (*P* < 0.05 or 0.01; Figures 3(a)–3(c)). Furthermore, immunofluorescence staining indicated that the fluorescence intensity of IL-6, IL-1*β*, and TNF-*α* was strong in the colonic tissues of DSS-induced mice, which were weakened in the YFBD (10 and 20 g/kg) and SASP groups (Figure 3(d)).

### 3.4. YFBD Decreased the Levels of Inflammatory Cytokines in LPS-Stimulated RAW264.7 Cells

**Figure 4:**
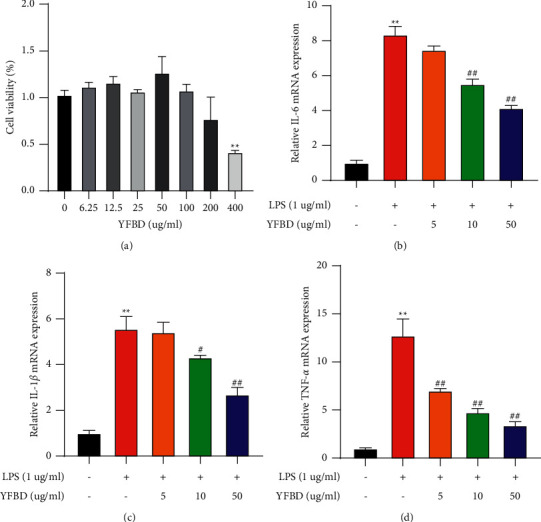
YFBD decreased the mRNA levels of inflammatory cytokines in LPS-induced RAW264.7 macrophages. (a) Effects of YFBD on the viability of RAW 264.7 macrophages. (b) mRNA level of IL-6. (c) mRNA level of IL-1*β*. (d) mRNA level of TNF-*α*. Data are presented as mean ± SEM (*n* = 3). ^*∗∗*^*P* < 0.01 vs. control; ^#^*P* < 0.05; ^##^*P* < 0.01 vs. LPS.

RAW264.7 cell models of experimental inflammation were used to further confirm the anti-inflammatory effects of YFBD [32, 33]. Cytotoxicity analysis indicated that the concentrations of YFBD used in the experiment had no significant influence on cell viability (Figure 4(a)). The mRNA levels of IL-6, IL-1*β*, and TNF-*α* in RAW264.7 cells increased strikingly after induction with LPS (*P* < 0.01; Figures 4(b)–4(d)), while the cytokines declined in the YFBD groups (10, 50 *μ*g/mL) compared with the DSS group (*P* < 0.05 or 0.01; Figures 4(b)–4(d)). Moreover, the study indicated that YFBD reduced the mRNA levels of IL-6, IL-1*β*, and TNF-*α* in a concentration-dependent manner.

### 3.5. YFBD Downregulated TLR4-Mediated PI3K/Akt and NF-*κ*B/p65 Pathways In Vivo

**Figure 5:**
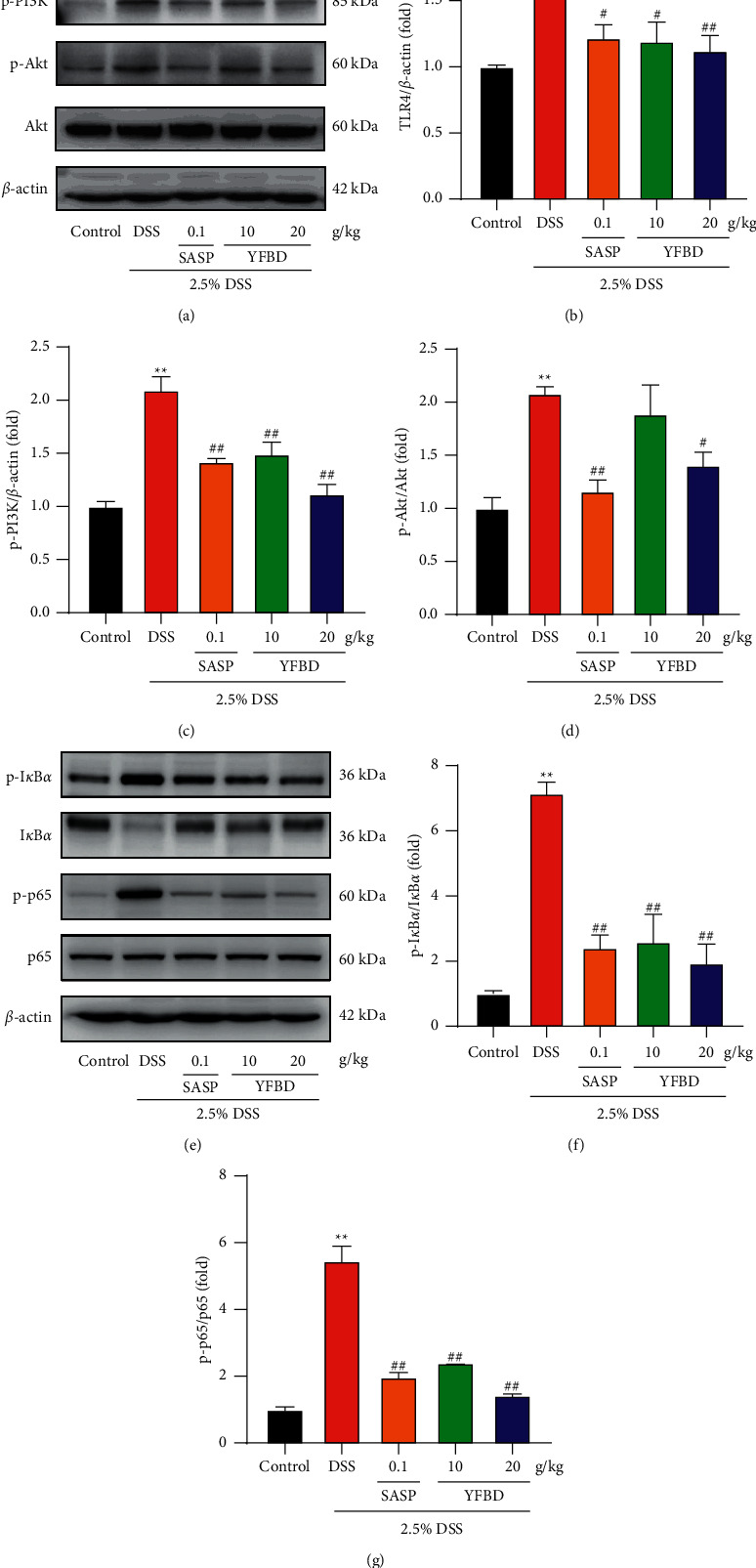
YFBD downregulated TLR4-mediated PI3K/Akt and NF-*κ*B pathways in colons of mice with DSS-induced colitis. (a) Effect of YFBD on the protein expression of TLR4, P-PI3K, and p-Akt. (b–d) Quantification of the ratio of TLR4, P-PI3K, and p-Akt. (e) Effect of YFBD on the degradation of I*κ*B*α* and phosphorylation of NF-*κ*B/p65. (f, g) Quantification of the ratio of I*κ*B*α* and phosphorylated NF-*κ*B/p65. Data are presented as mean ± SEM (*n* = 3). ^*∗∗*^*P* < 0.01 vs. control; ^#^*P* < 0.05; ^##^*P* < 0.01 vs. DSS.

TLR4-mediated PI3K/Akt and NF-*κ*B pathways play a vital role in regulating inflammatory responses. Therefore, this study evaluated the protein expression in the pathways using WB. In vivo, the expression of TLR4 increased significantly in the DSS group compared with the control group, as well as the elevated expression of p-PI3K, p-Akt, p-NF-*κ*B/p65, and p- I*κ*B*α* (*P* < 0.01; Figures 5(b)–5(g)). However, the expression of these proteins decreased in the YFBD (10, 20 g/kg) and SASP groups compared with the DSS group (*P* < 0.05 or 0.01; Figures 5(b)–5(g)).

### 3.6. YFBD Inhibited TLR4-Mediated PI3K/Akt and NF-*κ*B/p65 Pathways In Vitro

**Figure 6:**
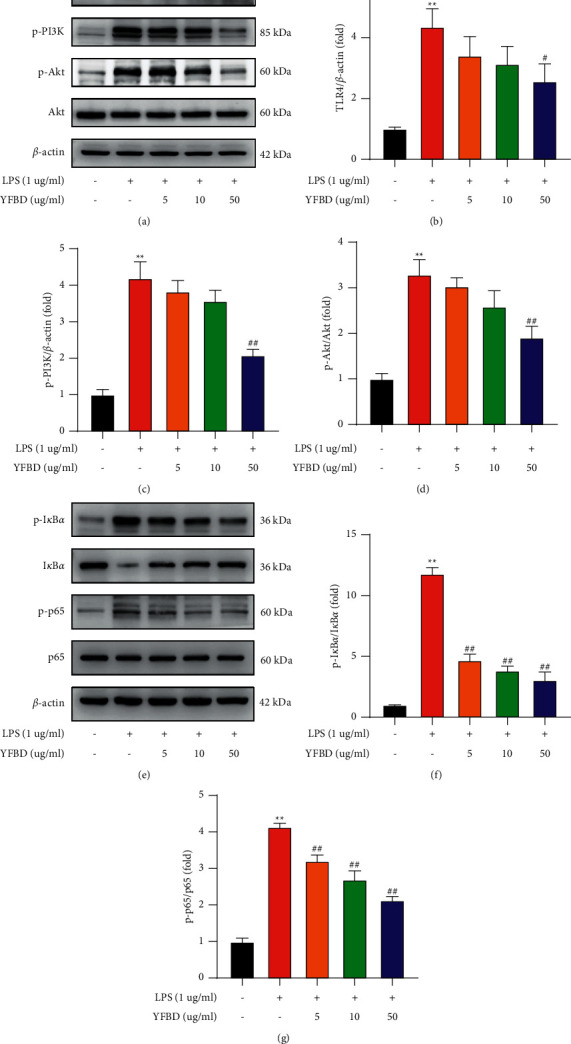
YFBD downregulated the TLR4-mediated PI3K/Akt and NF-*κ*B pathways in LPS-induced RAW264.7 cells. (a) Effect of YFBD on the protein expression of TLR4, P-PI3K, and p-Akt. (b–d) Quantification of the ratio of TLR4, P-PI3K, and p-Akt. (e) Effect of YFBD on the degradation of I*κ*B*α* and phosphorylation of NF-*κ*B/p65. (f, g) Quantification of the ratio of I*κ*B*α* and phosphorylated NF-*κ*B/p65. Data are presented as mean ± SEM (*n* = 3). ^*∗∗*^*P* < 0.01 vs. control; ^#^*P* < 0.05; ^##^*P* < 0.01 vs. LPS.

In vitro, LPS elevated the expression of TLR4, p-PI3K, p-Akt, p-NF-*κ*B/p65, and p-I*κ*B*α* in RAW264.7 cells (*P* < 0.01; Figures 6(b)–6(g)). The expression of TLR4, p-PI3K, and p-Akt showed no significant difference between LPS group and YFBD (5, 10 *μ*g/mL) groups (Figures 6(b)–6(d)), while they decreased significantly in YFBD (50 *μ*g/mL) group compared with LPS group (*P* < 0.05 or 0.01; Figures 6(b)–6(d)). The protein levels of p-NF-*κ*B/p65, and p-I*κ*B*α* were reduced markedly in different concentrations of YFBD in comparison with LPS group (*P* < 0.01; Figures 6(f) and 6(g)). The high-dose YFBD showed the most notable effects in the study.

**Figure 7:**
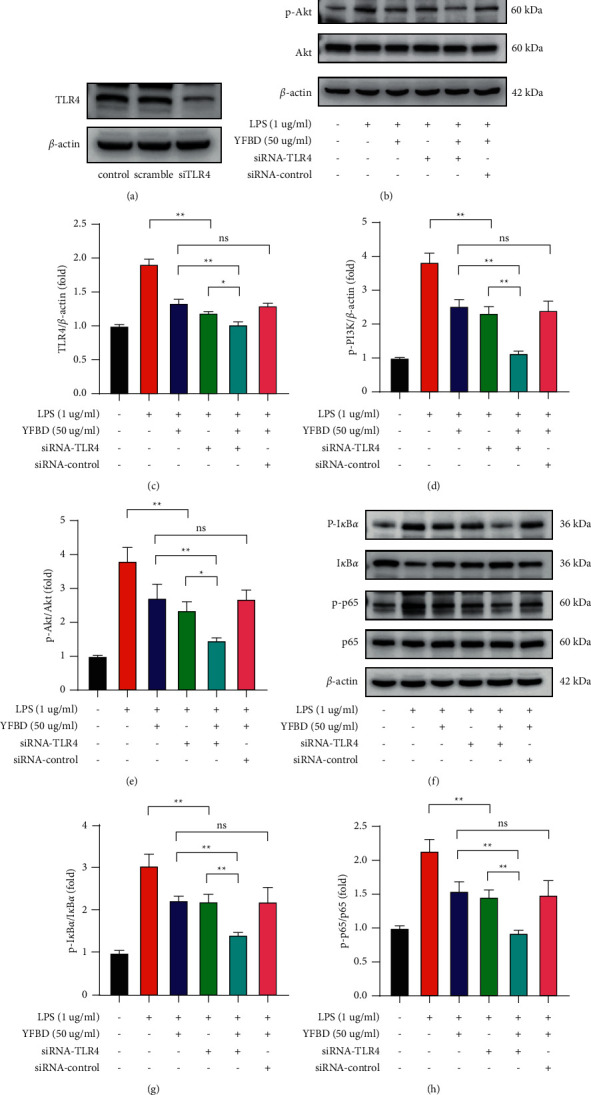
YFBD inhibited PI3K/Akt and NF-*κ*B pathways in RAW 264.7 cells via downregulating TLR4 expression. (a) RAW 264.7 cells were transfected with TLR4 siRNA or control siRNA for 48 h. (b) Effect of YFBD and TLR4 siRNA on the protein expression of TLR4, P-PI3K, and p-Akt. (c–e) Quantification of the ratio of TLR4, P-PI3K, and p-Akt. (f) Effect of YFBD and TLR4 siRNA on the degradation of I*κ*B*α* and phosphorylation of NF-*κ*B/p65. (g, h) Quantification of the ratio of I*κ*B*α* and phosphorylated NF-*κ*B/p65. Data are presented as mean ± SEM (*n* = 3). ^*∗∗*^*P* < 0.01; ^*∗*^*P* < 0.05.

To explore whether TLR4 is involved in the inhibition of YFBD on PI3K/Akt and NF-*κ*B pathways, we applied siRNA to knock down TLR4 in RAW 264.7 cells and detected the related proteins in the pathways. In this study, TLR4 was remarkably knocked down in siRNA transfected cells (Figure 7(a)). Meanwhile, TLR4-knockdown significantly downregulated the TLR4 expression in LPS-induced RAW 264.7 cells (*P* < 0.01; Figure 7(c)), and the additional YFBD treatment further reduced the TLR4 expression (*P* < 0.05; Figure 7(c)). Consistently, the phosphorylation of PI3K, Akt, NF-*κ*B/p65, and the degradation of I*κ*B*α* increased in LPS-induced cells, whereas TLR4-knockdown attenuated their phosphorylation or degradation (*P* < 0.01; Figures 7(d)–7(h)), which was similar to the effect of YFBD. Compared with the LPS-induced cells knocked down by siTLR4, the combination of YFBD and TLR4-knockdown decreased the expression of p-PI3K, p-Akt, p-NF-*κ*B/p65, and p-I*κ*B*α* to a greater extent (*P* < 0.05 or 0.01; Figures 7(d)–7(h)). This suggests that YFBD acts as an inhibitor of the pathway. Compared with the LPS-induced cells treated with YFBD, the additional interference of siTLR4 decreased the TLR4 expression and the protein expression in PI3K/Akt and NF-*κ*B pathways to the same extent (*P* < 0.01; Figures 7(c)–7(h)). The results indicated that the effects of YFBD on the inhibition of PI3K/Akt and NF-*κ*B pathways were at least partly through TLR4.

## 4. Discussion

UC is threatening the health of people all over the world due to its increasing incidence, undefined etiology, intractability, and recurrence [34]. TCM provides a wealth of weapons against this recurrent disease. This study confirmed the anti-inflammatory effects of YFBD on DSS-induced models, revealing initially that YFBD exerted its effects partly by regulating TLR4-mediated PI3K/Akt and NF-*κ*B pathways.

DSS-induced colitis closely imitates human UC in terms of both clinical manifestations and anatomical alterations [35]. Therefore, the model was used in the study. The results showed that YFBD could mitigate clinical symptoms and pathological changes in DSS-induced models. YFBD, as other prescriptions of TCM, is characterized by complex composition and action on multiple targets. HPLC analysis identified five effective constituents in YFBD: chlorogenic acid, caffeic acid, benzoylmesaconine, benzoyl aconitine, and quercetin. Most of these ingredients showed protective effects against experimental colitis [36–38], which cohered with the discovery of the current research. Furthermore, it was shown that high-dose YFBD had more superior curative effects compared with low-dose YFBD. On the one hand, low-dose YFBD could not protect against the invasion of DSS. On the other hand, when the low-dose group was exposed to worse colon injury, the blood flow tended to decrease, accompanied by the reduction in self-repairment ability. The two factors contributed to the significant discrepancy in curative effects between the two groups.

The mass accumulation of IL-6, IL-1*β* and TNF-*α* is one of the features of DSS-induced colitis, which amplifies the inflammatory cascades and accelerates the disease progression [39–41]. The serum levels of IL-6 are positively related to the clinical and histopathological severity of UC and can predict the possibility of clinical remission and relapse [42]. IL-1*β* can activate other cytokine targets, inducing inflammatory response synergistically [43]. The levels of IL-1*β* in tissues correlated with the disease activity of UC [44]. TNF-*α* plays a crucial role in the inflammatory response, and anti-TNF-*α* therapy has been shown as an effective approach [45]. Accordingly, the blocking of these cytokines is considered to be an effective strategy for treating UC. Therefore, the study further explored the influence of YFBD on maintaining the levels of inflammatory cytokines. The study revealed that YFBD decreased the levels of IL-1*β*, IL-6, and TNF-*α* both in vivo and in vitro. Interestingly, previous studies showed that the active ingredients of YFBD, including caffeic acid, chlorogenic acid, and quercetin, could also downregulate the levels of these cytokines in the colitis models [37, 38, 46, 47], which was in line with the present findings. Although the exact components of YFBD have not been identified, it is presumed that YFBD could achieve remarkable anti-inflammatory effects due to the combination of these ingredients.

**Figure 8:**
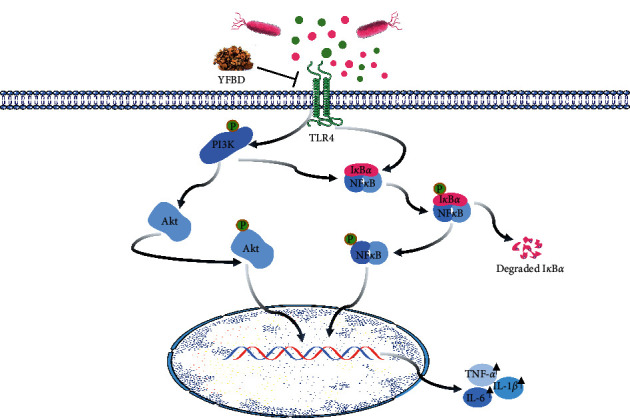
Schematic illustration depicting the potential pathways associated with the therapeutic effect of YFBD on ulcerative colitis.

Based on the aforementioned results, the study further explored the intracellular mechanism behind the anti-inflammatory properties of YFBD (Figure 8). DSS can destroy the mucosal barrier, which allows bacteria to invade the otherwise impermeable mucus [48]. LPS can be recognized by TLR4 [19, 20], and then, the TLR4-linked signaling cascade is initiated, including PI3K/Akt and NF-*κ*B pathways, which are related to inflammatory responses. NF-*κ*B can regulate the gene expression of several proinflammatory cytokines involved in UC pathogenesis [49]. Once activated, NF-*κ*B translocates to the nucleus and triggers the expression of various inflammatory genes. The PI3K/Akt pathway exerts its anti-inflammation effects through regulating the downstream molecules, such as m-TOR, which plays a central role in autophagy induction [50]. A previous study showed that activated m-TOR and impaired autophagy occupied a vital position in intestinal inflammation [51]. Meanwhile, the PI3K/Akt pathway can also enhance the production of proinflammatory cytokines by triggering the NF-*κ*B pathway. This study found that TLR4 was highly expressed in both animal and cell models, accompanied by increased expression of phosphorylated PI3K, Akt, NF-*κ*B/p65, and I*κ*B*α*. However, TLR4-mediated PI3K/Akt and NF-*κ*B pathways were downregulated with the treatment of YFBD. Meanwhile, the combination of YFBD and TLR4-knockdown decreased the expression of p-PI3K, p-Akt, p-NF-*κ*B/p65, and p-I*κ*B*α* to a greater extent compared with the LPS-induced cells knocked down by siTLR4 only. A previous research based on network pharmacology indicated that YFBD had positive effects on malignant tumors partly through PI3K and TNF pathways, which strengthened the evidence that YFBD could act on the pathways in our study [52]. These results demonstrated that the anti-inflammatory activity of YFBD was probably mediated by the inactivation of TLR4-mediated PI3K/Akt and NF-*κ*B signaling pathways and YFBD might play the role of TLR4 inhibitor in treating UC.

However, this study had limitations. First, the signaling pathways related to UC are multiple, and YFBD might have multiple targets in treating UC. Apart from TLR4-mediated PI3K/Akt and NF-*κ*B pathways, other pathways might participate in treating UC simultaneously, which were not examined in this study. Hence, future studies need to explore and clarify the involvement of the other pathways. Second, the components in YFBD are multiple and complex, and the active components involved in treating UC remain unclear. Next, we plan to extract the main active ingredients in YFBD and investigate the effects of these ingredients in treating UC through further experiments.

## 5. Conclusions

In summary, YFBD decreased the production of inflammatory cytokines in DSS-induced colitis and LPS-stimulated RAW 264.7 cells. It exerted anti-inflammatory effects via inactivating TLR4-mediated PI3K/Akt and NF-*κ*B pathways.

## Figures and Tables

**Table 1:** Description of YFBD.

Latin name	Chinese name	English name	Origin	Amount (g)	Batch codes
*Coix lacryma-jobi* L.	Yi Ren	Semen coicis	Guizhou	30	20150136
*Radix Aconiti Lateralis Preparata*	Fuzi	Monkshood	Sichuan	6	20160240
*Patrinia scabiosaefolia* Fisch.	Bai Jiang Cao	Patrinia	Jiangsu	15	20160240

**Table 2:** Primers for qRT-PCR.

Genes	Primer sequences (5′-3′)	Length (bp)
IL-6	F: CACTTCACAAGTCGGAGGCT	113
	R: CTGCAAGTGCATCATCGTTGT	

IL-1*β*	F: AACCTTTGACCTGGGCTGTC	144
	R: AAGGTCCACGGGAAAGACAC	

TNF-*α*	F: AGTTCTATGGCCCAGACCCT	149
	R: ACAAGGTACAACCCATCGGC	

*β*-actin	F: CGCCACCAGTTCGCCATGGA	105
	R: TACAGCCCGGGGAGCATCGT	

## Data Availability

The data used to support the findings of this study are available from the corresponding author upon request.
